# SKIL facilitates tumorigenesis and immune escape of NSCLC via upregulating TAZ/autophagy axis

**DOI:** 10.1038/s41419-020-03200-7

**Published:** 2020-12-02

**Authors:** Fang Ma, Meng-Ge Ding, Yi-Yu Lei, Li-Hua Luo, Shun Jiang, Yu-Hua Feng, Xian-Ling Liu

**Affiliations:** 1 Department of Oncology, The Second Xiangya Hospital, Central South University, 410011 Changsha, Hunan Province P.R. China; 2 Cancer Center, Union Hospital, Tongji Medical College, Huazhong University of Science and Technology, 430022 Wuhan, P.R. China

**Keywords:** Immunology, Cancer

## Abstract

Immune escape is an important mechanism in tumorigenesis. The aim of this study was to investigate roles of SKIL in tumorigenesis and immune escape of non-small-cell lung cancer (NSCLC). SKIL expression levels in NSCLC cell line, clinical sample, and adjacent normal tissue were measured by quantitative PCR, western blot, or immunohistochemistry. Lentivirus was used to overexpress/silence SKIL or TAZ expression. Malignant phenotypes of NSCLC cells were evaluated by colony formation, transwell, and MTT assays, and in xenograft mice model. Syngeneic mice model and flow cytometry were used to evaluate T cell infiltration. Quantitative PCR and western blot were applied to evaluate relevant mRNA and protein levels, respectively. Co-immunoprecipitation was applied to unveil the interaction between SKIL and TAZ. SKIL expression was higher in NSCLC tissue compared to adjacent normal tissue. Silencing of SKIL inhibited malignant phenotypes of NSCLC cells and promoted T cell infiltration. SKIL-knockdown inhibited autophagy and activated the STING pathway in NSCLC cells through down-regulation of TAZ. Silencing of TAZ cancelled the effects of SKIL overexpression on malignant phenotypes and autophagy of NSCLC cells. Inhibition of autophagy reversed the effects of SKIL/TAZ overexpression on the STING pathway. In conclusion, SKIL promoted tumorigenesis and immune escape of NSCLC cells through upregulation of TAZ/autophagy axis and inhibition on downstream STING pathway.

## Introduction

Non-small-cell lung cancer (NSCLC) remains a major global health threat, which affects 1.8 million people and causes an estimated 1.59 million death worldwide^1^. Although currently there are several available treatments for NSCLC (e.g. surgery, radiotherapy, chemotherapy, and targeted therapy), the prognosis of NSCLC patients is still unsatisfactory^2^. In the past few years, immunotherapy has emerged as a new treatment method for cancer. Immune system plays important roles in eliminating cancer cells before they can form clinically identifiable tumor. In order to survive this immune pressure, some of the cancer cells develop a way to escape the surveillance of immune system (so-called immune escape), mainly through interacting with immune checkpoint molecules expressed on regulatory T cells (e.g. cytotoxic T-lymphocyte antigen-4 (CTLA-4) and programmed death-1 (PD-1)) and subsequent inhibition of the activation of regulatory T cells^3^. Immunotherapy targeting those immune checkpoint molecules has been intensively studied and achieved success in clinical trials in NSCLC^4^.

Cyclic GMP–AMP (cGAMP) synthase (cGAS) and stimulator of interferon (IFN) gene (*STING*) were reported to play important roles in anti-cancer immunity, e.g. T cell immunity^5–9^. cGAS is a key cytosolic DNA sensor. When cytosolic DNA (e.g. microbial DNA) is detected, cGAS produces cGAMP which promotes the translocation of STING from endoplasmic reticulum and Golgi apparatus to perinuclear vesicles^10,11^. Re-located STING could activate TANK-binding kinase 1 (TBK1), NF-κB, and interferon regulatory transcription factor 3 (IRF3), which then stimulate the production of IFN, an essential factor in anti-cancer immunity^12^, and other inflammatory cytokines^10^. Previous studies reported that inhibiting DNA damage response proteins (poly-ADP-ribose polymerase (PARP) and checkpoint kinase 1 (CHK1)) could activate cGAS-STING-mediated anti-cancer immunity, and enhance the blockade of PD-1 checkpoint and infiltration of cytotoxic T cell^13,14^. Those findings potentiate the cGAS–STING pathway as a therapeutic target to enhance the efficacy of PD-1 immune checkpoint inhibitors.

Firstly discovered as a proto-oncogene, SKIL (also known as SnoN) is a mediator of the transforming growth factor-β (TGF-β) signaling pathway and exerts pro-oncogenic activity through inhibiting TGF-β/Smad pathway^15^. Overexpression of SKIL was reported in esophageal squamous cell carcinoma (ESCC)^16^ and breast cancer^17^, and amplification of SKIL was also found in NSCLC, prostate cancer, and head and neck squamous carcinoma (HNSC)^18^. In addition, SKIL was involved in reduction of autophagy and subsequent tumor cell apoptosis in arsenic trioxide-treated promyelocytic leukemia^19^ and ovarian carcinoma^20^. Evidence also showed that expression of SKIL was elevated in BCL11A-overexpressed lung squamous carcinoma^21^. On the other hand, some studies suggested suppressive roles of SKIL on the proliferation and survival in some specific pathological subtypes of cancer (e.g. lower grade esophageal adenocarcinoma, lower grade ovarian adenocarcinoma, pancreatic adenocarcinoma, and breast ductal adenocarcinoma^22^), and silencing of SKIL (or SnoN) upregulated proliferation of ESCC cell line^23^, indicating a complex of SKIL in tumorigenesis. In addition, no study has reported role of SKIL in immune escape.

YAP/TAZ is closely-related transcriptional regulators and plays important roles in many biological processes, e.g. organ growth, tissue biology, and cancer^24,25^. In addition, activation of YAP/TAZ was found in several types of cancer and associated with cancer proliferation, progression, and metastasis^26,27^. YAP/TAZ receives input from several signaling pathways, including Hippo pathway, Wnt pathway, and F-actin^24^. For example, as important effector of the Hippo pathway, YAP/TAZ could be phosphorylated and inhibited by key members of Hippo complex, Lats2 and Salvador (Sav)^28,29^. On the other hand, YAP/TAZ regulates many downstream signaling factors, e.g. connective tissue growth factor (CTGF), cysteine-rich angiogenic inducer 61 (CYR61), and modulates interactions between cells and extracellular matrix^30^. Similar to SKIL^19,20^, YAP/TAZ was also reported to assist cancer cells to bypass the normal contact inhibition by promoting autophagy through regulation on expression of myosin-II and F-actin stress fibers^31^, which pathway is commonly compromised in normal noncancerous cells. Interestingly, a recent study reported that SKIL promoted the tumorigenesis of breast cancer by enhancing the activity of TAZ^32^, which might be an important mechanism for the oncogenic effects of SKIL.

In our study, we investigated the relationship between SKIL and TAZ, and their functions on autophagy and malignant phenotype of NSCLC. We found that SKIL induced malignant phenotypes of NSCLC through upregulation of TAZ pathway and autophagy, which also led to inhibition on immune escape from T cell immunity through inhibition on the STING pathway.

## Methods

### Sample collection and ethics

Human NSCLC and paired adjacent normal tissue samples were collected from patients who received surgery from 2017 to 2019 in Department of Oncology, The Second Xiangya Hospital, Central South University. After removal of tumor, blocks of tumor tissue (1 cm × 1 cm × 0.2 cm) and adjacent normal tissue were collected and put into formalin-fixed and paraffin-embedded (FFPE) blocks following standard procedure in pathology department, or frozen at −80 °C for future analysis. Informed consent was obtained from each patient enrolled in our study. Our study protocol was approved by Institutional Ethics Committee of Central South University.

### Cell culture

Human lung adenocarcinoma cell lines (HCC827, CALU-3, NCI-H1975), human lung carcinoma cell line (A549), human lung squamous cell carcinoma cell lines (NCI-H226, NCI-H520, SK-MES-1), human bronchial epithelial cell line (16HBE) which was immortalized from normal bronchial epithelial cells and retains their characteristic features, and anaplastic murine lung carcinoma cell line (Madison 109 lung carcinoma, M109) were obtained from Fenghui Biology (Hunan, China). All the cell lines have passed the STR authentication. After thawing, cell lines were cultured in RPMI-1640 culture medium supplemented with 10% fetal bovine serum and 1% penicillin/streptomycin. Cells were kept in humid atmosphere with 5% carbon dioxide at 37 °C. Culture medium was changed every other day and cells were sub-cultured when reached 80% confluence. All the cultured cells were routinely checked for mycoplasma contamination.

### Animal model

To evaluate the in vivo tumorigenesis of cell lines, BALB/c-nu nude mice were obtained from Department of Laboratory Animals, Central South University, divided randomly into two groups, injected subcutaneously with 0.5 × 10^6^ of tumor cells each. Size of tumor was monitored every 4 days using a caliper. Tumor volume was calculated by (tumor length × tumor width^2^)/2. On day 24, mice were euthanized by carbon dioxide and tumor blocks were collected from the animals for subsequent analysis. For syngeneic mice model, mouse lung cancer cell line (M109) and BALB/c mice were used instead, and 0.5 × 10^6^ of cultured M109 cells were injected subcutaneously into each BALB/c mouse with Matrigel (1:1; BD Biosciences, USA). On day 21, mice were euthanized by carbon dioxide and tumor blocks were collected. All procedures were approved by Department of laboratory animals, Central South University. The investigators were blinded to the group allocation during the experiment and when assessing the outcome.

### Flow cytometry

The collected tumor blocks were firstly minced into pieces and single-cell suspension were obtained by passing the tumor mince through a cell strainer (Corning, USA). After fixation and permeabilization using BD Cytofix/Cytoperm kit (554714, BD Biosciences), cells were stained with anti-CD3 (100246, Biolegend), anti-CD4 (100526, Biolegend), anti-CD45 (101917, Biolegend), or anti-CD8 (100722, Biolegend) antibody following standard flow cytometry protocol. Results were then obtained using Fortessa platform (BD Biosciences) and analyzed using FlowJo software (BD Biosciences). CD3 and CD45 were used to analyze total T cells (both CD45 and CD3 positive). Cytotoxic T cells were further analyzed using CD4 and CD8 after gating of CD3^+^ cells, while cells from inguinal lymph node were used as reference of gating.

### Western blot

Tissue or cell samples were homogenized in 1× phosphate-buffered saline (PBS). Protein concentration of the samples was determined using Pierce Rapid Gold BCA Protein Assay Kit (Themo Fisher, USA). One microgram of protein from each sample was loaded onto SDS-PAGE gel. After separation of the proteins using electrophoresis and transferring onto nitrocellulose membranes, targeted proteins were blocked in 5% (w/v) skimmed milk in TBST. Proteins were then stained at 4 °C overnight with specific primary antibody: SNAIL1 (#3895, CST), SLUG (#9585, CST), E-cadherin (#14472, CST), vimentin (#3932, CST), GAPDH (#97166, CST), STING (#13647, CST), TBK1 (#3504, CST), p-TBK1 (#5483, CST), IRF3 (#4302, CST), p-IRF3 (#29047, CST), β-actin (#58169, CST), Beclin-1 (#3738, CST), p62 (#39749, CST), LC3-I (#4599, CST), LC3-II (#2775, CST), TAZ (#4883, CST), CTGF (#10095, CST), CYR61 (#39382, CST), LATS2 (#5888, CST), Sav (#13301, CST), or p-TAZ (#59971, CST). On the next day, membranes were washed in 1× PBS, and cultured with anti-mouse IgG (#7076, CST) or anti-rabbit IgG (#7074, CST) secondary antibody depending on the type of primary antibody for 2 h at room temperature. Results were then visualized using gel-imaging system (GS-800, Bio-Rad, USA), followed by densitometric analysis.

### Quantitative PCR

Total RNA was extracted using TRIzol reagents (Invitrogen, USA) following the manufacturer’s instruction. Frozen tissue samples were thawed at room temperature. After thawing, 50–100 mg tissue sample was homogenized in 1 ml of TRIzol reagent. For cell samples, 1 × 10^6^ cells were lysed in 1 ml TRIzol reagent. After adding 0.2 ml chloroform, sample homogenate was incubated for 2 min and centrifuged for 15 min at 12,000 × *g*, 4 °C. The upper aqueous phase was transferred into a new tube and mixed with equal volume of 70% ethanol. The samples were filtered into a spin cartridge by centrifugation and the flow-through was discarded. The RNAs were then eluted using RNase-free water. After determination of RNA concentration by measuring absorbance of diluted samples at 260 and 280 nm using a spectrophotometer (NanoDrop, Thermo Scientific, USA), 1 μg RNA from each sample was used for reverse transcription. Quantitative PCR (qPCR) was performed using SYBR Select Master Mix (Invitrogen, USA) on ABI 7500 system (ABI, USA) following a standard procedure. Specific primers were purchased from Sangon Biotech (Shanghai, China). Detailed sequences for each primer pair are: human CXCL10, sense GTGGCATTCAAGGAGTACCTC, antisense TGATGGCCTTCGATTCTGGATT; human CCL5, sense CCAGCAGTCGTCTTTGTCAC, antisense CTCTGGGTTGGCACACACTT; human IFN-β, sense CATTACCTGAAGGCCAAGGA, antisense CAATTGTCCAGTCCCAGAGG; mouse CXCL10, sense CCCACGTGTTGAGATCATTG, antisense GTGTGTGCGTGGCTTCACT; mouse CCL5, sense ATATGGCTCGGACACCACTC, antisense TCCTTCGAGTGACAAACACG; SOX2, sense CCCACCTACAGCATGTCCTACTC, antisense TGGAGTGGGAGGAAGAGGTAAC; SKIL, sense ACCAGTTATTATTCCCCTGTTCCT, antisense GGCATGGCTTACCAGAAACC; CD44, sense CCAGAAGGAACAGTGGTTTGGC, antisense ACTGTCCTCTGGGCTTGGTGTT; OCT3/4, sense CCTGAAGCAGAAGAGGATCACC, antisense AAAGCGGCAGATGGTCGTTTGG; CD133, sense CACTACCAAGGACAAGGCGTTC, antisense CAACGCCTCTTTGGTCTCCTTG; NANOG, sense CTCCAACATCCTGAACCTCAGC, antisense CGTCACACCATTGCTATTCTTCG.

### Transfection

For overexpression of *SKIL* and *TAZ* genes, full coding region of target gene (*human SKIL, mouse SKIL, human TAZ*, or *mouse TAZ*) was amplified and cloned into lentivirus vector pLVX-IRES-Neo. For knockdown of *SKIL* and *TAZ* genes, short hairpin RNA (shRNA) was purchased (Sangon Biotech, China) and cloned into pLVX-IRES-Neo. Empty pLVX-IRES-Neo vector was used as control. The lentivirus vectors were then used for the transfection of target cells. The transfection was performed using Lipofectamine 2000 system (Thermo Fisher, USA) following the manufacturer’s instruction. Cells were seeded in a six-well plate with packaging medium at 70–80% confluence and allowed to incubate overnight at 37 °C in humidified atmosphere with 5% CO_2_. On the next day, cells were transfected with lentivirus vectors and incubated at 37 °C in humidified atmosphere with 5% CO_2_. Packaging medium was carefully replaced 6 h after the transfection. Forty-eight hours after the transfection, cells with stable transfection were selected using culture medium containing 1.5 μg/ml puromycin (Sigma-Aldrich, USA). After selection, culture medium was changed and cells with stable transfection were used for subsequent treatment and analysis.

### MTT assay

3-(4,5-Dimethylthiazol-2-yl)-2,5-diphenyltetrazolium bromide (MTT) assay was performed to evaluate the viability of cells. Briefly, after suspension in culture medium, cultured cells were mixed with equal volume of 5 mg/ml MTT (M2128, Sigma, dissolved in 1× PBS) and incubated at 37 °C for 1 h. After removing medium, 200 μl DMSO was used to suspend MTT metabolic product. Mixture was incubated at 37 °C for 10 min, and optical density (OD) was measured at 490 nm.

### Colony formation assay

Briefly, cultured cells were trypsinzed and suspended in culture medium. Four thousand cells were then suspended in culture medium containing 0.4% low-melting-point agarose (Sigma, USA), which was overlaid on hardened 1.2% agarose bottom layer in 60 mm dishes. After cooling, the dishes were incubated at 37 °C in humidified atmosphere with 5% CO_2_. Culture medium was changed every 3 days. On day 14, colonies were stained with 1% crystal violet, and number of colonies which were larger than 200 μm was counted under a light microscope (Leica Microsystems, USA) and recorded.

### Transwell assay

Transwell assay was performed to evaluate the migration and invasion ability of cells. Transwell inserts suitable for 24-well plates (8.0 μm pores, Corning, USA) were used. For cell invasion ability analysis, the down side of the transwell membrane on the inserts was coated with Matrigel (Corning, USA, diluted in cold DMEM) at 4 °C, and incubated at 37 °C for 30 min to allow gelling. After reaching 50–60% confluence, culture cells were trypsinized and suspended in culture medium. Cell suspension was placed into upper chamber of the insert with Matrigel, and the insert was put into a sterile 24-well plate containing 500 μl culture medium in each well. For cell migration ability analysis, the re-suspended cells were placed in to upper chamber without Matrigel. After incubation for 24 h in humidified atmosphere with 5% CO_2_, cells on the upper side of the insert membrane was completely removed using cotton swab. Inserts were fixed using 4% polyfluoroalkoxy and stained with 1% crystal violet. Invasion or migration ability of cells was evaluated by number of cells attached to the lower side of the insert. Quantification of the cells was performed by imaging of the insert membranes and subsequent analysis using ImageJ.

### Co-immunoprecipitation

Immunoprecipitation was performed according to Zhu et al.^32^. Briefly, high-salt lysis buffer was prepared using 420 mM NaCl, 50 mM HEPES-KOH (pH 7.8), 5 mM EDTA, 0.1% NP-40, 3 mM dithiothreitol (Sigma-Aldrich, USA), 0.5 mM PMSF (Sigma-Aldrich, USA), and 10 μg/ml aprotinin (Sigma-Aldrich, USA). Cells were lysed in high-salt lysis buffer. Before the immunoprecipitation, cell lysates were cleared using protein A-Sepharose beads (Proteintech, IL, USA). Protein A-Sepharose beads coupled with anti-SKIL antibody (19218-1-AP, Proteintech, IL, USA) were then used to precipitate endogenous SKIL in cell lysates. Precipitated proteins were subject to further western blot analysis.

### Immunofluoresence staining

Cells were firstly let grow on coverslips. Before staining, cells were fixed in 4% formaldehyde in 10% methanol for 20 min. After blocking with 10% goat serum, cells were cultured with diluted LC3B primary antibody (#12741, CST, USA) for 1 h, and then secondary goat anti-rabbit IgG antibody conjugated with Alexa Fluor 594 (#8889, CST, USA) for 1 h at room temperature. After washing with 1 × PBS, cells were stained with ProLong Gold Antifade Mountant with DAPI (P36935, Themo Fisher, USA). Result was quickly visualized under a fluorescence microscope (Leica Microsystems, USA) with excitation at 561 nm.

### Immunohistochemistry

Tumor and normal tissue were fixed in formalin for 24 h, and then dehydrated and embedded in paraffin following standard pathology procedure in pathology department. The FFPE blocks were then sectioned at 4 μm and stored at 4 °C. On the day of staining, slides were de-paraffinized in xylene followed by ethanol, and then hydrated in de-ionized water. For antigen-retrieval, slides were heated in Tris-EDTA buffer (pH 9.0) in a pressure cooker for 3 min at full pressure and allowed to cool down in running tap water for 10 min. After washing with 1× PBS, slides were stained with anti-SKIL primary antibody (1:50, ab78979, abcam, USA) for 1 h, and then HRP-conjugated anti-rabbit secondary antibody (1:1000, ab6721, Abcam, USA). Staining result was visualized using 3,3′-diaminobenzidine (DAB, D8001, Sigma-Aldrich, USA) under a light microscope (Leica Microsystems, USA).

### Statistical analysis

Statistical analysis was performed using GraphPad Prism 7.0. Data meet normal distribution. Variance was similar between the groups that are being statistically compared. Student’s *t-*test was used to compare between two groups, while one-way ANOVA were used for multiple group comparison. *P* < 0.05 was considered statistically significant.

## Results

### SKIL and TAZ expression levels are higher in NSCLC tissue

**Fig. 1:**
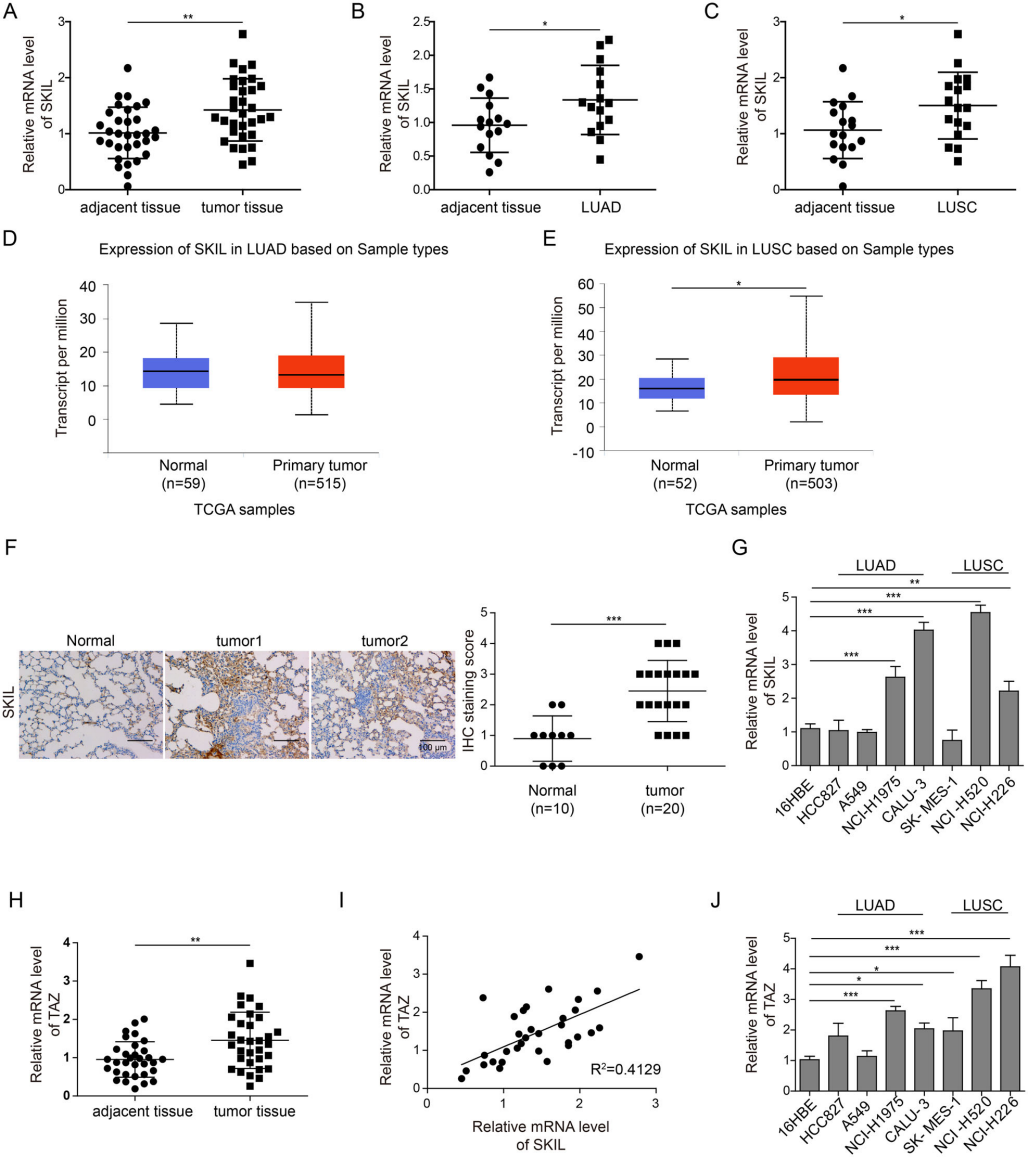
Expression of SKIL in NSCLC tissue. qPCR measurement showed that SKIL expression was significantly higher in **a** NSCLC tissue (*n* = 32), **b** lung adenocarcinoma (LUAD) (*n* = 15), and **c** lung squamous carcinoma (LUSC) (*n* = 17), compared to adjacent tissue. Analysis on TCGA samples showed that SKIL expression was higher in **d** LUSC samples but not in **e** LUAD samples, compared to adjacent tissue. **f** Immunohistochemistry results showed increased SKIL protein expression in NSCLC compared to adjacent normal tissue. **g** SKIL mRNA expression levels were elevated in NCI-H1975, CALU-3 (both are LUAD), NCI-H520, and NCI-H226 (both are LUSC) cell lines, but not in other lung cancer cell lines screened (HCC827, A549, and SK-MES-1), compared to control normal lung cell line (16HBE). **h** TAZ mRNA expression was higher in tumor tissue compared to adjacent normal tissue. **i** Positive correlation was found between the relative expression levels of SKIL and relative expression of TAZ in lung cancer tissue. **j** TAZ expression was significantly higher in NCI-H226, NCI-H520, SK-MES-1, NCI-H1975, and HCC827 lung cancer cell lines compared to control cell line (16HBE), which was not observed in CALU-3 and A549 cell lines. *P < 0.05, **P < 0.01, ***P < 0.001. Experiments were performed in triplicate.

In all, we collected tumor and adjacent tissue samples from 32 NSCLC patients, of which 15 patients had lung adenocarcinoma (LUAD) and 17 had squamous carcinoma (LUSC). Measurement of SKIL expression using qPCR and IHC showed that SKIL expression was significantly higher in NSCLC tissue (Fig. 1a, f). Analysis using western blot showed increased SKIL protein expression in NSCLC tumor tissue compared to adjacent normal tissue, and a positive correlation was found between SKIL mRNA and protein expression levels in tumor (Fig. S1A, B). Further subgroup analysis showed that SKIL expression was significantly higher in LUSC and LUAD tissue compared to adjacent normal tissue (*P* < 0.05, see Fig. 1b, c). We also investigated SKIL expression of NSCLC samples using TCGA database, and obtained similar results in LUSC (Fig. 1e) but not in LUAD (Fig. 1d). Screening of SKIL expression levels in lung cancer cell lines showed that SKIL mRNA expression was elevated in some of LUAD cell lines (NCI-H1975 and CALU-3) and LUSC cell lines (NCI-H520 and NCI-H226), compared to normal lung cell line (16HBE), while SKIL level was not elevated in HCC827, A549, and SK-MES-1 cell lines (Fig. 1g). Western blot results showed that SKIL protein expression was elevated in HCC827, A549, NCI-H1975, CALU-3, NCI-H520, and NCI-H226 cell lines, compared to control. Correlation analysis also showed a positive correlation between SKIL mRNA and protein expression levels in human lung cancer cell lines (Fig. S1C, D). Measurement of TAZ expression using qPCR also revealed increased TAZ mRNA levels in NSCLC tumor tissue compared to adjacent normal tissue (Fig. 1h). Correlation analysis showed a positive correlation between the relative SKIL expression levels and TAZ expression levels in tumor tissue of NSCLC patients (Fig. 1i). Results of lung cancer cell lines showed significantly higher TAZ expression levels in NCI-H226, NCI-H520, SK-MES-1, NCI-H1975, and HCC827 compared to control (16HBE), which was not seen in CALU-3 and A549 cell lines (Fig. 1j). This trend was different from the observations in SKIL expression in lung cancer cell lines (Fig. 1g). Overall, those results indicate that SKIL and TAZ expression levels are elevated in NSCLC. Based on the screening results from cell lines, CALU-3 (LUAD) and NCI-H520 (LUSC) which showed high expression of SKIL were chosen for subsequent analysis.

### Silencing on SKIL expression inhibited malignant phenotype and in vivo growth of CALU-3 and NCI-H520

**Fig. 2:**
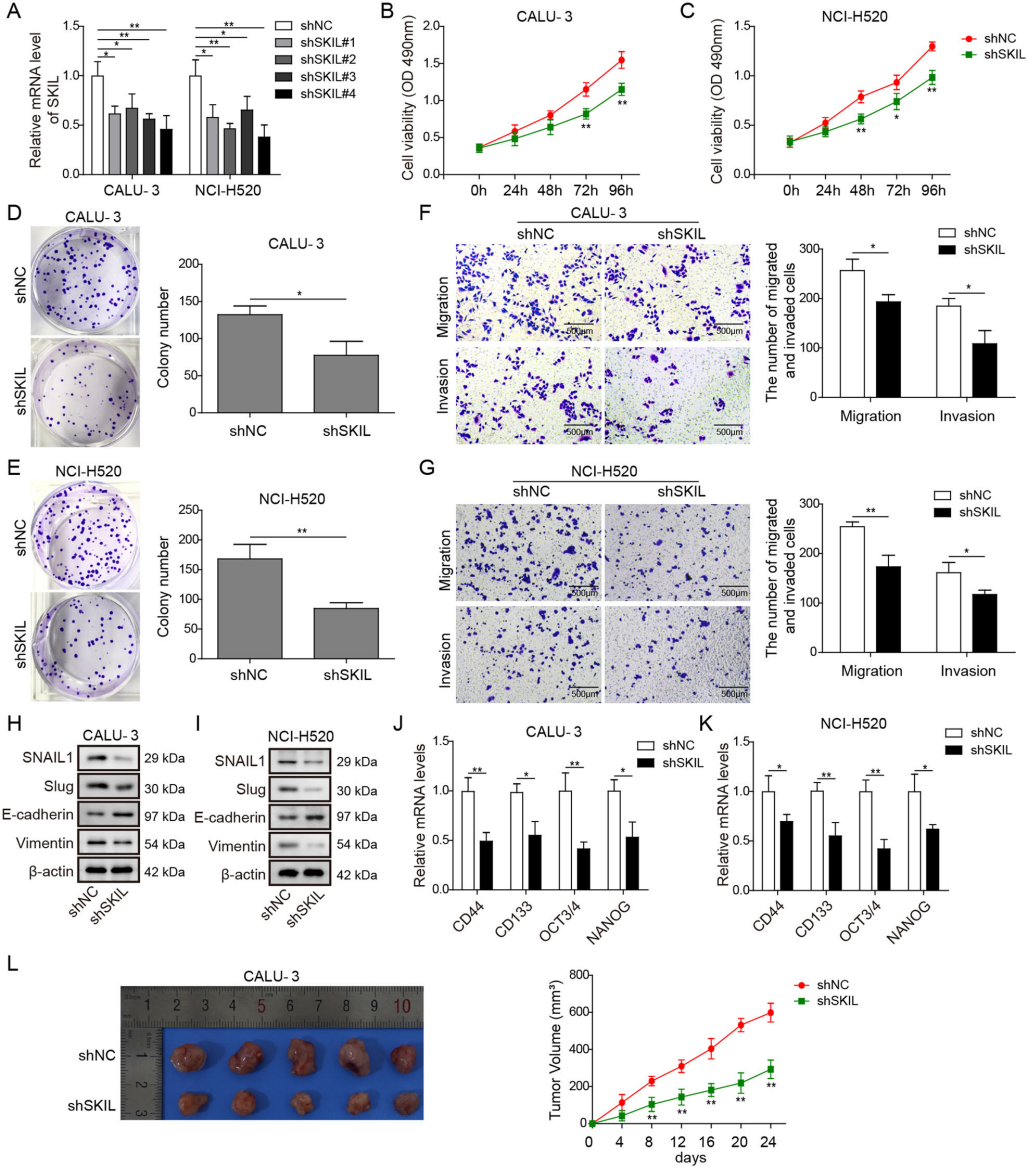
Silencing of SKIL inhibited malignant phenotype of NSCLC. **a** After silencing of SKIL in CALU-3 and NCI-H520 cell lines using shRNA, SKIL expression was significantly inhibited in all the four shRNAs (shSKIL#1–4) compared to control shRNA (shNC), while shSKIL#4 showed the lowest SKIL mRNA expression level. **b**, **c** MTT assay showed inhibited viability after SKIL was silenced (shSKIL) in CALU-3 and NCI-H520 compared to control (shNC). **d**, **e** Colony formation assay showed fewer colonies formed in CALU-3 and NCI-H520 with silenced SKIL expression (shSKIL) compared to control (shNC). **f**, **g** Transwell assay showed inhibited cell migration and invasion in the shSKIL group compared to the control group (shNC). **h**, **i** Western blot measurement showed decreased SNAIL1, SLUG, and vimentin expression levels, and increased E-cadherin levels in shSKIL group (shSKIL), compared to control group (shNC) in both CALU-3 and NCI-H520. **j**, **k** qPCR results showed that mRNA levels of cancer stem cell markers (CD44, CD133, SOX2, OCT3/4, and NANOG) were decreased after SKIL silencing (shSKIL) compared to control (shNC). **l** Tumorigenesis assay in nude mice using CALU-3 and NCI-H520 showed slower tumor growth rate and smaller size of collected tumor blocks after animal sacrifice in the shSKIL group (shSKIL) compared to the control group (shNC). *P < 0.05, **P < 0.01. Experiments were performed in triplicate.

In order to understand the relationship between SKIL and malignant phenotype of NSCLC, we silenced SKIL in CALU-3 and NCI-H520. Based on qPCR results, we selected shSKIL#4 for subsequent analysis, which showed the most significant knockdown on SKIL expression in both cell lines (Fig. 2a). In addition, complementation experiment using another shRNA (shSKIL#2) showed that SKIL overexpression could reverse the effects of shSKIL#2 on cell viability, migration, invasion, and epithelial–mesenchymal transition (EMT) of NCI-H520 cell line (Fig. S3).

After SKIL was silenced by the shRNA we chose (shSKIL#4) (see qPCR and western blot results in Fig. 2a and S1E), CALU-3 and NCI-H520 showed significantly decreased viability, colony formation (proliferation), invasion, and migration abilities (Fig. 2b–g). Measurement of EMT markers showed decreased expression of SNAIL1, SLUG, and vimentin, and increased expression of E-cadherin in SKIL-silenced CALU-3 and NCI-H520 (Fig. 2h, i), indicating inhibited EMT in those cells. Expression levels of cancer stem cell markers (CD44, CD133, SOX2, OCT3/4, and NANOG) were also decreased after silencing of SKIL in CALU-3 and NCI-H520 (Fig. 2j, k). Overall, silencing of SKIL resulted in inhibited malignant phenotypes (viability, proliferation, invasion, migration, EMT, and cancer stem cell viability) in CALU-3 and NCI-H520. Furthermore, we also conducted in vivo xenograft experiments, and results showed a reduced tumor growth in xenograft generated from SKIL-silenced CALU-3 cells (Fig. 2l).

### Silencing on SKIL expression increased T cell infiltration and release of chemokines via activation on the STING pathway

**Fig. 3:**
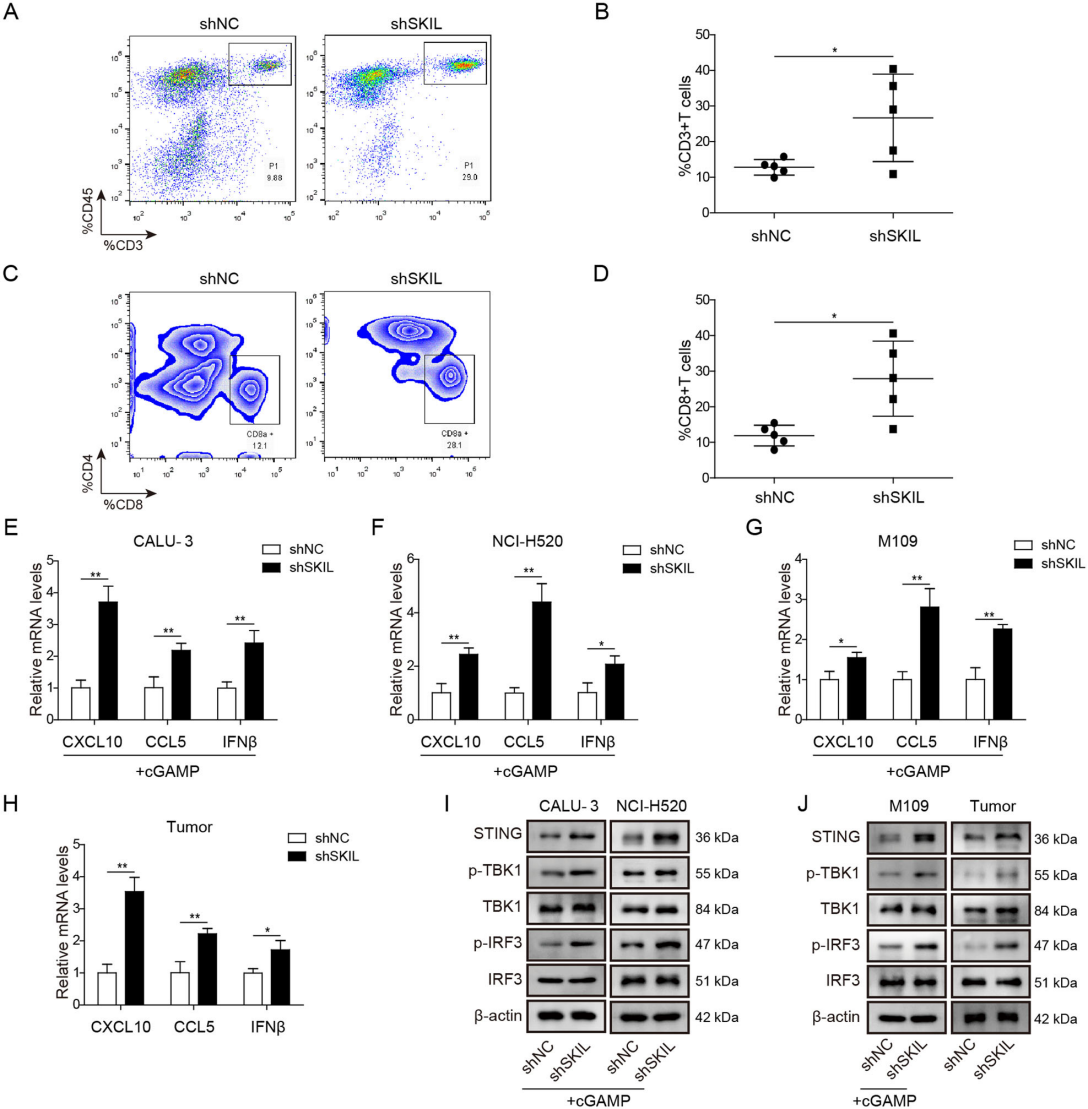
SKIL silencing promoted T cell infiltration through activation of STING pathway. After subcutaneous injection of NSCLC cells into BALB/c mice, tumor blocks were collected on day 24, and flow cytometry analysis showed significantly increased number of **a**, **b** total T cells and **c**, **d** cytotoxic T cells in SKIL-silenced NSCLC cells (shSKIL) compared to control (shNC). qPCR showed that SKIL silencing significantly increased mRNA levels of CXCL12, CCL5, and IFN-β in **e** CALU-3, **f** NCI-H520, and **g** M109 cell lines and **h** tumor blocks. **i**, **j** Western blot analysis results showed increased expression levels of STING, p-TBK1, and p-IRF3 in CALU-3, NCI-H520, M109 cell lines, and tumor blocks. *P < 0.05, **P < 0.01. Experiments were performed in triplicate.

In addition to malignant phenotypes, we further investigated the influence of SKIL on T cell-related immune escape of tumor cells. A mouse-derived lung cancer cell line (M109) was treated with shSKIL (or control shRNA) and used to generate tumor in immunocompetent BALB/c mice (synergic model). After 21 days, mice were euthanized and tumor blocks were collected. Flow cytometry showed increased number of total T cell (CD3^+^ CD45^+^) and cytotoxic T cell (CD3^+^ CD45^+^ CD8^+^) in the SKIL-silenced xenograft model (Fig. 3a–d) compared to control. qPCR analysis showed that levels of chemokines (CXCL10, CCL5, and IFN-β) which are involved in cytotoxic T cell recruitment were elevated in SKIL-silenced CALU-3 and NCI-H520 at the presence of STING pathway agonist (cGAMP) and in SKIL-silenced xenograft model (Fig. 3e–h), indicating an activation of IFN-β pathway. Expression levels of STING were increased when SKIL was silenced, together with increased phosphorylation of STING downstream signaling pathway factors, including TBK1 and transcription factor interferon regulatory factor 3 (IRF3)^33^ in CALU-3 and NCI-H520 at the presence of cGAMP and in SKIL-silenced xenograft model (Fig. 3i, j), indicating that knockdown of SKIL might influence T cell infiltration and release of relevant chemokines through activation of the STING pathway.

### Silencing on SKIL expression inhibited autophagy and attenuated TAZ activation

**Fig. 4:**
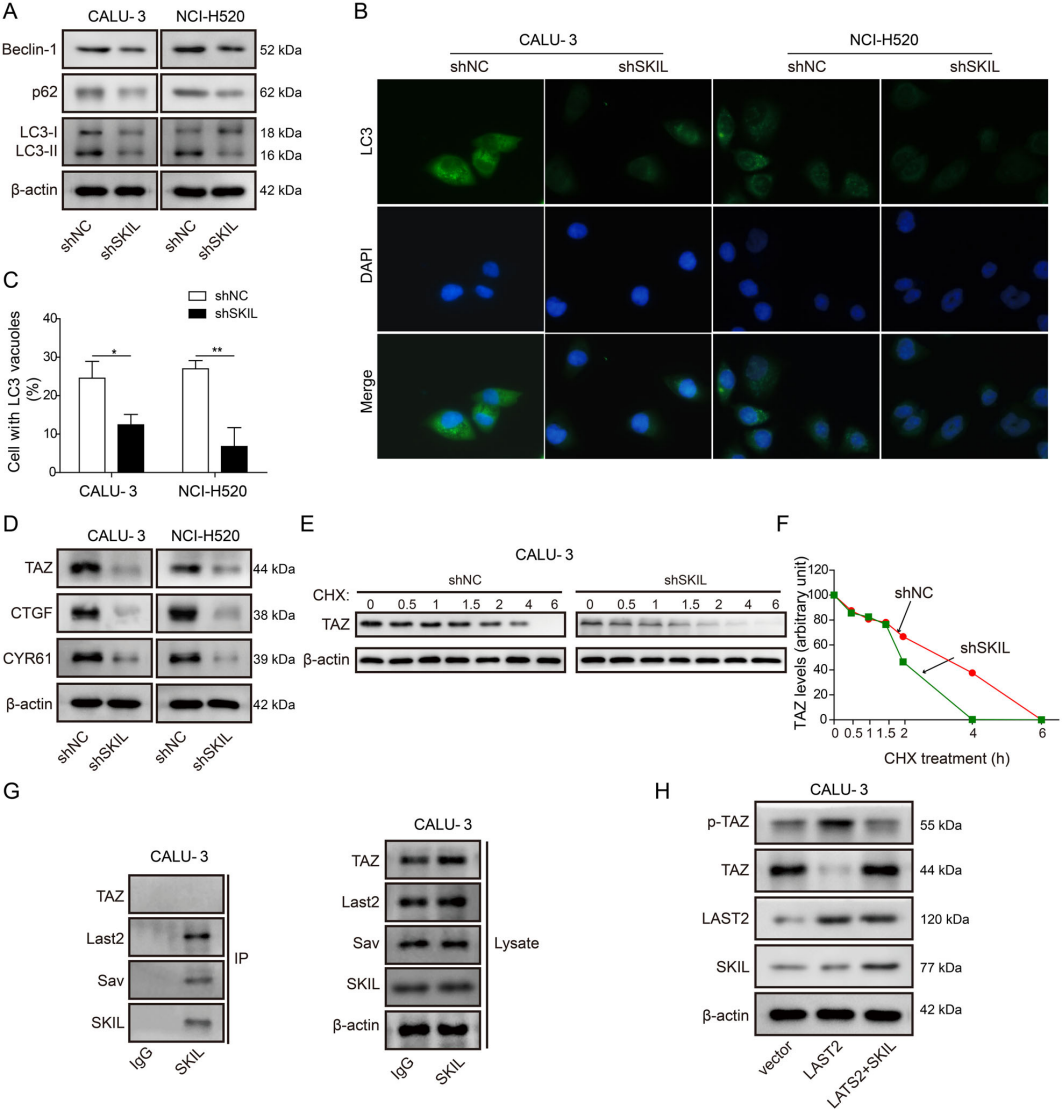
SKIL induced autophagy through inhibition on TAZ phosphorylation by LATS2 and degradation. **a** Western blot analysis showed that SKIL silencing decreased expression levels of autophagy markers (LC3, p62, and Beclin-1). **b**, **c** Immunofluorescence staining indicated significant decrease of autophagosome after SKIL silencing in both CALU-3 and NCI-H520 cell lines. Percentage of cells with LC3 vacuoles were counted and calculated from 100 cells in 20 fields **d** Western blot analysis showed that SKIL silencing decreased expression of TAZ and its downstream signaling factors (CTGF, CYR61). **e**, **f** Cycloheximide treatment resulted in faster TAZ protein degradation in SKIL-silenced CALU-3 (shSKIL) compared to control (shNC). **g** Co-immunoprecipitation assay showed that SKIL could bind to elements of Hippo complex (LATS2, Sav), but not TAZ. **h** Western blot analysis showed that overexpression of LATS2 led to increased phosphorylation of TAZ (p-TAZ) and decreased levels of TAZ. Further overexpression of SKIL reversed the effect of LATS2 overexpression on levels of p-TAZ and TAZ, without influencing LATS2 level. *P < 0.05, **P < 0.01. Experiments were performed in triplicate.

Since SKIL was reported to regulate autophagy in several types of cancer^19,20^, we also investigated the relationship between SKIL and autophagy in NSCLC. Measurement on autophagy-related markers (LC3-II/I, p62, and Beclin-1) showed decreased levels of those markers in SKIL-silenced CALU-3 and NCI-H520 (Fig. 4a). Formation of autophagosome was also inhibited in SKIL-silenced cell lines, indicated by decrease of cells with LC3-positive vacuoles (Fig. 4b, c). Previous study found that SKIL could promote tumorigenesis of breast cancer through enhancement of TAZ signaling^32^. In addition, TAZ was reported to regulate autophagy in different types of cancer^31,32^. Therefore, we also measured levels of TAZ and its downstream pro-tumorigenesis factors, and results showed decreased levels of TAZ and its downstream factors (CTGF, CYR61) in SKIL-silenced cell lines (Fig. 4d), suggesting that SKIL might regulate autophagy via regulation on the TAZ pathway in NSCLC.

We further investigated the possible interaction between SKIL and TAZ. Results showed that SKIL silencing accelerated TAZ degradation when treated with cycloheximide (Fig. 4e, f). Co-immunoprecipitation (co-IP) results showed that SKIL could bind to elements of hippo complex (LATS2 and Salvador, Sav), but not TAZ (Fig. 4g). Overexpression of LATS2, an inhibitor of TAZ^32^, promoted degradation of TAZ through phosphorylation, while SKIL overexpression inhibited phosphorylation and degradation of TAZ without influencing the level of LATS2 (Fig. 4h). Those results suggest that SKIL could indirectly regulate TAZ activation through interaction with LATS2, which might be involved in the regulation of SKIL on autophagy in NSCLC.

### TAZ is involved in the induction of autophagy and malignant phenotype of NSCLC cells by SKIL

**Fig. 5:**
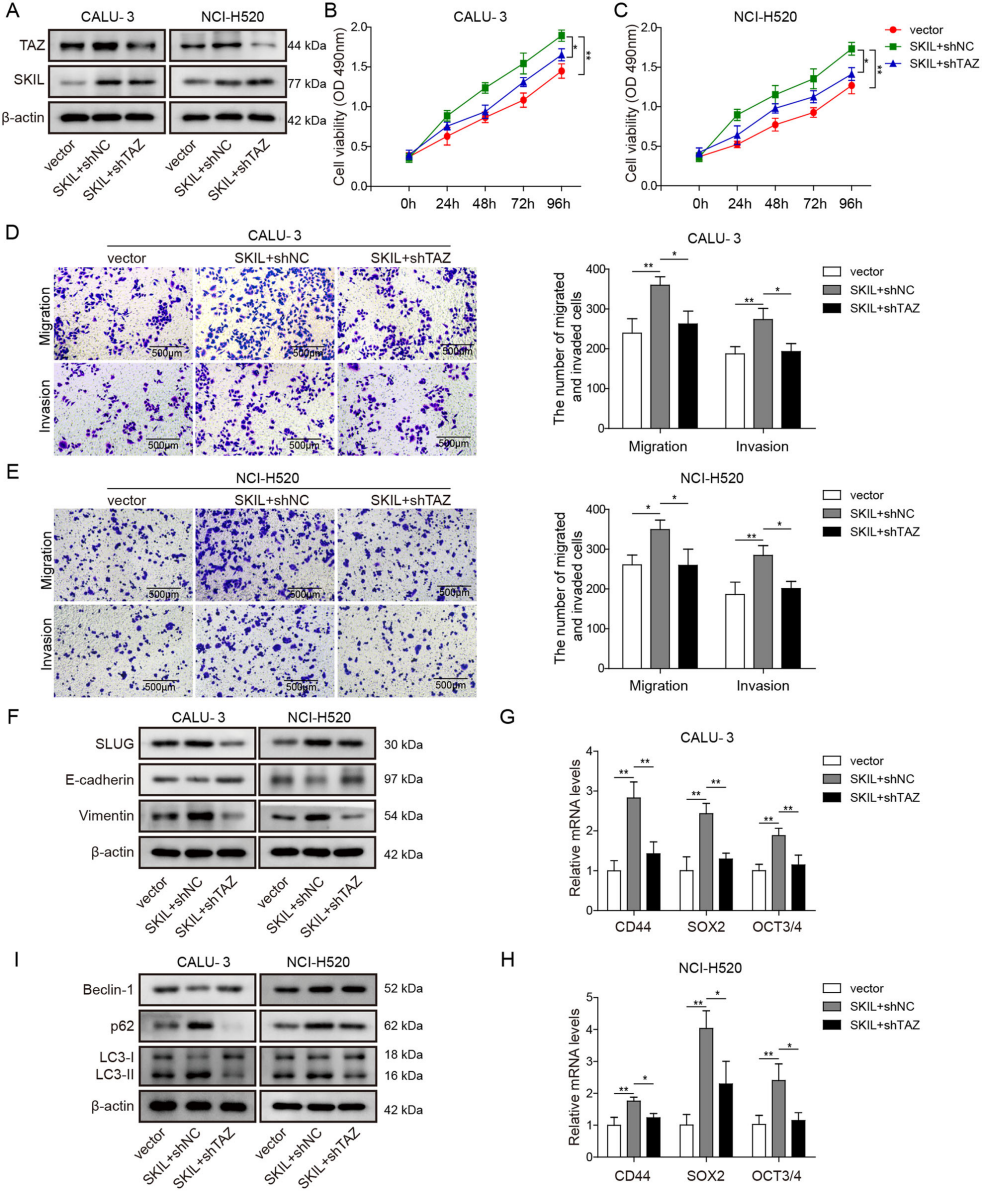
TAZ was involved in SKIL-induced autophagy and tumorigenesis. **a** Western blot analysis showed increased SKIL expression after transfection of vectors carrying SKIL gene (SKIL), and further transfection of shRNA targeting TAZ (shTAZ) decreased TAZ expression. SKIL overexpression in CALU-3 and NCI-H520 (SKIL + shNC) increased **b**, **c** cell viability and **d**, **e** ability of migration and invasion compared to control (vector), which was reversed by further silencing of TAZ (SKIL + shTAZ) in those cells. **f** Western blot analysis on EMT-related factors showed that SKIL overexpression increased levels of SLUG and vimentin, and decreased levels of E-cadherin in both CALU-3 and NCI-H520, which was reversed by TAZ silencing. Western blot or qPCR measurement showed that levels of **g, h** cancer stem cell markers (CD44, SOX2, OCT3/4) and **i** autophagy-related proteins (LC3, p62, Beclin-1) were increased by SKIL overexpression, which was reversed by further silencing of TAZ in those cells. *P < 0.05, **P < 0.01. Experiments were performed in triplicate.

To further demonstrate the role of TAZ in the relationship between SKIL and malignant phenotype and autophagy of NSCLC cell, we used vectors to overexpress SKIL in CALU-3 and NCI-H520 and then silenced TAZ expression in those cells (Fig. 5a). Overexpression of SKIL significantly increased cell viability, and invasion and migration abilities in CALU-3 and NCI-H520, while further silencing of TAZ led to decreased cell viability, and migration and invasion abilities compared to SKIL overexpression group (Fig. 5b–e), indicating that TAZ plays key roles in the regulation of malignant phenotype by SKIL. Furthermore, overexpression of SKIL increased expression of SLUG and vimentin, decreased E-cadherin expression, as well as increased levels of cancer stem cell markers (CD44, SOX2, OCT3/4) and autophagy markers (LC3, p62, Beclin-1), while further TAZ silencing reversed the effect of SKIL on those markers compared to SKIL overexpression group (Fig. 5f–i). All those results indicate key roles of TAZ in the regulation of NSCLC malignant phenotype and autophagy by SKIL.

### TAZ is involved in the regulation of T cell infiltration and release of chemokines by SKIL

After silencing TAZ in M109 cell line, we transplanted those cells into BALB/c mice. Tumor blocks were collected after euthanizing mice using carbon dioxide on day 21. Flow cytometry showed increased numbers of total T cell and cytotoxic T cell (Fig. S4A–D) in tumor blocks generated by TAZ-silenced M109 cells. qPCR results showed that levels of chemokines (CXCL10, CCL5, IFN-β) were increased after silencing of TAZ in CALU-3, NCI-H520, and M109 at the presence of STING pathway agonist cGAMP and in SKIL-silenced NSCLC cell-derived tumor blocks (Fig. S4E–H). Subsequent analysis showed that levels of STING and phosphorylation of its pathway downstream factors (p-TBK1, p-IRF3) were elevated when TAZ was silenced (Fig. S4I, J). Those effects after TAZ silencing were similar to that of SKIL silencing (Fig. 3a, j), indicating that TAZ may be involved in the regulation of T cell infiltration and release of chemokines by SKIL through STING pathway.

**Fig. 6:**
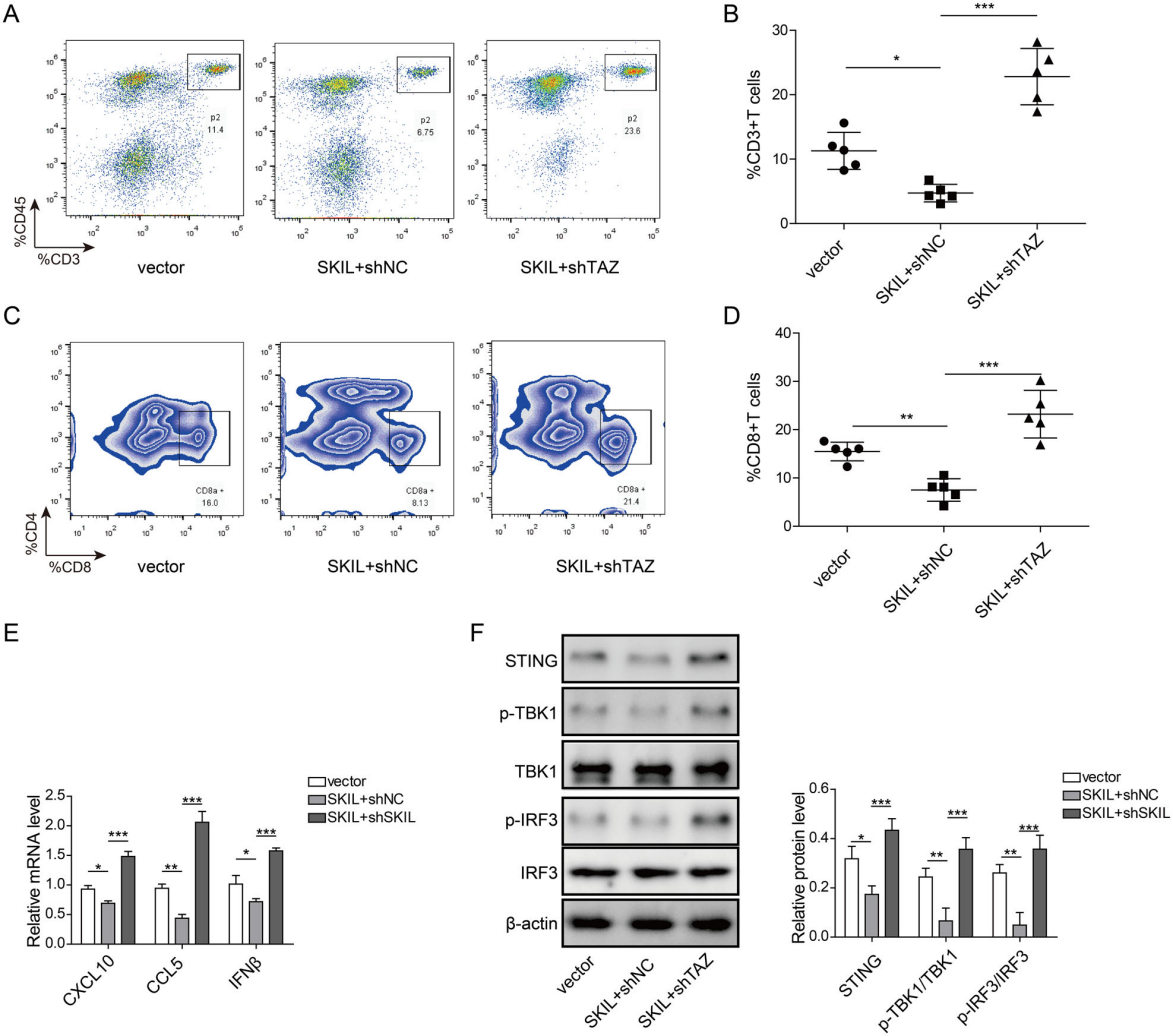
TAZ silencing promoted T cell infiltration in SKIL-overexpressed tumors through activation of STING pathway. M109 cell line with SKIL overexpression, with both SKIL overexpression and TAZ silencing, or control M109 mice lung cancer cell line were injected subcutaneously into BALB/c mice. On day 21, mice were euthanized and tumor blocks were collected. **a**, **b** Flow cytometry showed lower number of total T cells (CD45^+^CD3^+^) in SKIL-overexpressed tumor compared to control group. Tumors with both SKIL overexpression and TAZ silencing showed significantly higher number of total T cells, compared to tumors with SKIL overexpression. **c**, **d** Flow cytometry analysis showed decreased cytotoxic T cells (CD4^+^CD8^+^) in tumors with SKIL overexpression, compared to control. Further silencing of TAZ in those cells significantly increased numbers of cytotoxic T cells in those tumors, compared to tumors with SKIL overexpression. **e** SKIL-overexpressed tumors showed lower levels of CXCL10, CCL5, and IFN-β compared to control. Tumors with both SKIL-overexpression and TAZ silencing showed significantly higher levels of CXCL10, CCL5, and IFN-β, compared to SKIL-overexpression group. **f** Expression levels of STING, p-TBK1, and p-IRF3 were decreased in SKIL-overexpressed tumors compared to control tumor. Tumors with both SKIL overexpression and TAZ silencing showed significantly higher STING expression and levels of p-TBK1 and p-IRF3 compared to tumors with SKIL overexpression only. *P < 0.05, **P < 0.01. Experiments were performed in triplicate.

In order to further convince the role of SKIL/TAZ axis in immune escape, we overexpressed SKIL in M109 cells. In those cells, we further silenced the expression of TAZ. Those cells were transplanted into BALB/c mice and tumor blocks were collected after 21 days. After examination of flow cytometry, compared to control, SKIL-overexpressed tumor showed lower numbers of total T cells and cytotoxic T cells. Further silencing of TAZ significantly increased the numbers of total and cytotoxic T cells in tumor blocks, compared to SKIL-overexpression group (Fig. 6a–d). In addition, levels of chemokines (CXCL10, CCL5, IFN-β) were lower in SKIL-overexpressed tumor compared to control, and further TAZ silencing significantly increased chemokine levels in those SKIL-overexpressed tumor (Fig. 6e). Analysis on the expression levels of STING pathway showed that SKIL overexpression decreased STING levels and phosphorylation of its downstream factors (p-TBK1, p-IRF3), compared to control. Further silencing of TAZ significantly increased the levels of STING, p-TBK1, and p-IRF3, compared to SKIL-overexpression group (Fig. 6f). Those findings further demonstrated the important roles of SKIL/TAZ axis in immune escape through regulation on STING pathway.

### TAZ and autophagy are involved in the regulation of T cell infiltration and release of chemokines by SKIL

**Fig. 7:**
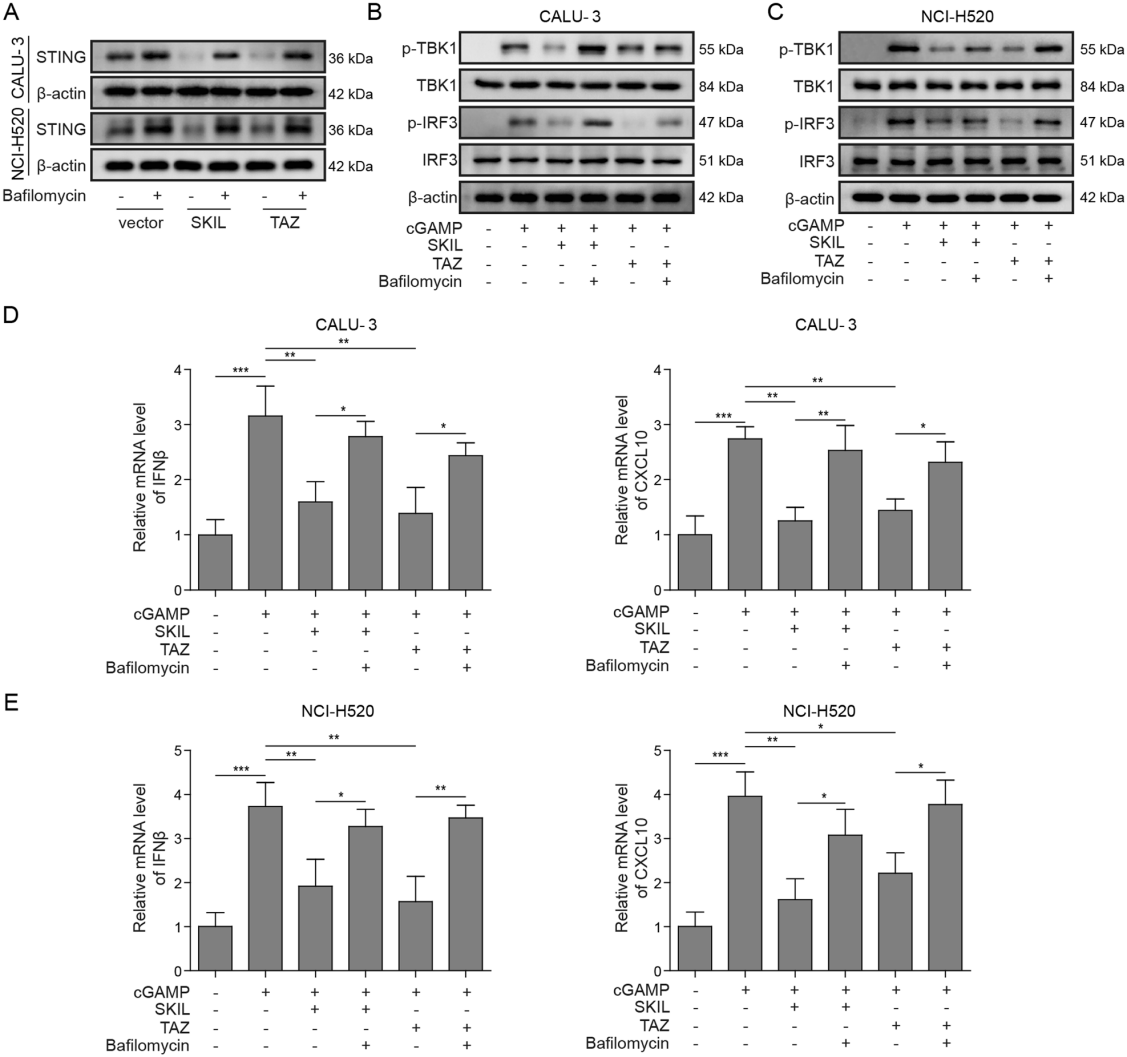
Autophagy was involved in the regulation of SKIL/TAZ on STING signaling pathway. **a** Western blot result showed that overexpression of either SKIL or TAZ decreased expression of STING in CALU-3 and NCI-H520, which was reversed by inhibition on autophagy using autophagy inhibitor (bafilomycin). **b**, **c** In CALU-3 and NCI-H520, treatment of cGAMP, a STING pathway agonist, resulted in increased p-TBK1 and p-IRF3. SKIL or TAZ overexpression decreased levels of p-TBK1 and p-IRF3, which was canceled by bafilomycin. **d**, **e** In CALU-3 and NCI-H520, SKIL or TAZ overexpression significantly inhibited cGAMP-induced expression of IFN-β and CXCL10, while treatment of bafilomycin canceled the inhibition. *P < 0.05, **P < 0.01, ***P < 0.001. Experiments were performed in triplicate.

Instead of silencing SKIL or TAZ, we also overexpressed the expression of those two factors and used an autophagy inhibitor, bafilomycin, to treat CALU-3 and NCI-H520. Using cGAMP to activate STING pathway, both SKIL and TAZ overexpression resulted in decreased STING levels, which could be canceled by inhibition on autophagy using bafilomycin (Fig. 7a). Similarly, overexpression of SKIL and TAZ inhibited STING pathway activation (decreased levels of p-TBK1, p-IRF3) and expression of chemokines (CXCL10, IFN-β), whereas inhibition on autophagy could cancel the inhibition (Fig. 7b–e). Those results suggest that autophagy was involved in the regulation on STING pathway and T cell activation by SKIL and TAZ.

## Discussion

Our study found that SKIL expression was increased in NSCLC and was associated with its malignant phenotypes and immune escape from T cell immunity. Further investigation on the underlying mechanism indicated that the promotion of malignant phenotype was through upregulation of TAZ by interaction with Hippo complex factors (LATS2) and subsequent activation of autophagy. The enhanced autophagy also inhibited STING pathway and activation of T cells, leading to increased immune escape.

Findings of previous studies indicated complex roles of SKIL in tumorigenesis. Expression of SKIL was elevated in many types of cancer^16–18,34^, but was also found to be inhibited in some subtypes of esophageal, ovarian, pancreatic, and breast cancer^22,23^. Chromosome region 3q26, which contains *SKIL* gene, was found to be amplified in 31.2% of NSCLC^18^, and similarly, our results showed that SKIL expression was elevated in tumor samples of NSCLC patients (see Fig. 1). In addition, for the first time, we demonstrated that SKIL could induce malignant phenotype and immune escape in NSCLC (Figs. 2 and 3). Further investigation showed that those effects of SKIL were dependent on upregulation of TAZ expression by interaction between SKIL and Hippo complex elements, Lats2 and Sav (Figs. 4 and 5). This finding was consistent with previous findings that SKIL (SnoN) promoted TAZ signaling through antagonizing Hippo complex in breast cancer^32^. TAZ was found to be over-activated in most types of cancer and is important for tumorigenesis^30^. Through upregulation on downstream factors (amphiregulin, CTGF and CYR61), TAZ could induce many malignant phenotypes of cancer, e.g., cancer stem cell viability, EMT, increased migration and invasion abilities, and higher potential of metastasis^30^. On the other hand, YAP/TAZ was reported to induce autophagy, and this YAP/TAZ–autophagy axis was involved in contact inhibition between normal cells, which was abnormally activated in cancer cells^31^. Our study results showed that SKIL induced autophagy in NSCLC cell lines, and TAZ was essential in this induction (Fig. 5i). Together, those findings indicate that in addition to TAZ signaling pathway (CTGF, CYR61), SKIL may also induce malignant phenotype of NSCLC cells through TAZ–autophagy axis.

Autophagy is a highly conserved cellular mechanism to sequestrate and degrade damaged cytoplasmic structures, which plays key roles in management of cell stress and survival^35^. Defected autophagy is commonly observed in cancer, and is involved in many malignant phenotypes of tumor, including motility, invasion, cancer stem cell viability, EMT, metastasis, etc.^35^. Previous studies also found roles of autophagy in immune response (e.g. affecting release of cytokines, survival, and activation of lymphoid cells), and defects in autophagy could promote immune escape of developing tumors^36^. SOX2 was found to induce escape of HNSC cells from T cell immunity through autophagy-dependent inhibition on STING pathway^37^. Similarly, our results showed that SKIL induced immune escape of NSCLC cells from T cell immunity through autophagy-dependent inhibition on STING pathway (Fig. 7), and TAZ was involved in the activation of autophagy in this signaling pathway (Fig. S4). Considering the previous findings that TAZ could regulate expression of SOX2 (ref. ^38^) which could also contribute to the regulation of autophagy and STING pathway^37^, and p62 (another critical autophagy-related protein) could attenuate STING pathway^39^, the SKIL/TAZ-induced inhibition on STING pathway may be mediated by multiple signaling pathways. On the other hand, STING was reported to induce autophagy, which is important in clearance of DNA and viruses in cytoplasm^40^. The induction of autophagy by STING was thought to be a primordial function of cGAS pathway, but may also be considered as a feedback mechanism for the regulation of STING pathway by autophagy. More investigations are required to further clarify their relationship.

In summary, our study showed that SKIL expression was increased in NSCLC, and SKIL promoted malignant phenotypes of NSCLC cells via TAZ-dependent upregulation of autophagy. In addition, to the best of our knowledge, our study is the first to show roles of SKIL in immune escape of cancer cells, and to illustrate the underlying mechanisms which involved upregulation of TAZ and autophagy, and subsequent inhibition on STING pathway. Further studies are needed to clarify more details in roles of SKIL in tumorigenesis and underlying molecular mechanisms.

## Supplementary information

Supplementary material

FigS1

FigS1

FigS3

FigS4

FigS5

FigS6

FigS7
