# Supplementary material for: SKIL facilitates tumorigenesis and immune escape of NSCLC via upregulating TAZ/autophagy axis
Source: Cell Death Dis. 2020 Dec 2;11(12):1028. doi: 10.1038/s41419-020-03200-7 (PMC7710697; doi:10.1038/s41419-020-03200-7)
Supplement: Supplementary file 1 — Supplementary material [file 41419_2020_3200_MOESM1_ESM.docx]

**Figure S1 SKIL protein expression in tumor and cell lines, and correlation with SKIL mRNA levels. (A)** Western blot results showed increased SKIL protein expression in tumor tissue compared to adjacent normal tissue. **(B)** Correlation analysis showed positive correlation between SKIL mRNA expression and SKIL protein expression. **(C)** Western blot analysis showed higher SKIL protein expression levels in HCC827, A549, NCI-H1975, CALU-3, NCI-H520, and NCI-H226 cell lines, compared to 16HBE cell line. SKIL protein expression level was lower in SK-MES-1 cell line, compared to 16HBE. **(D)** Positive correlation was found between SKIL mRNA and protein expression levels in cell lines. **(E)** Western blot analysis showed decreased SKIL protein levels in SKIL-knockdown cells. **P* < 0.05, ***P* < 0.01, ****P* < 0.001. Experiments were performed in triplicate.

**Figure S2 shSKIL#4 sufficiently inhibited SKIL expression in CALU-3 and NCI-H520 cell lines. (A)** Western blot analysis showed decreased SKIL expression in CALU-3 cells transfected with shSKIL#4, compared to control CALU-3 cells transfected with shNC. **(B)** Western blot analysis showed decreased SKIL expression in NCI-H520 cells transfected with shSKIL#4, compared to control NCI-H520 cells transfected with shNC. **P* < 0.05, ***P* < 0.01, ****P* < 0.001. Experiments were performed in triplicate.

**Figure S3 Effect of other shRNA on cell lines. (A)** MTT assay showed that NCI-H520 cell line transfected with shSKIL#2 (shSKIL#2) had decreased cell viability compared to control group (shNC). **(B)** NCI-H520 cell line transfected with shSKIL#2 (shSKIL#2) showed decreased migration and invasion abilities compared to cells transfected with control shRNA (shNC). **(C)** Western blot analysis showed increased E-cadherin and decreased vimentin levels in NCI-H520 cell line transfected with shSKIL#2 (shSKIL#2) compared to control (shNC). **(D)** SKIL expression was decreased in NCI-H520 cells transfected with shSKIL#2 and an empty lentivirus vector (shSKIL#2+ox-vector) compared to control cells transfected with control shRNA (shNC); in NCI-H520 cells transfected with both shSKIL#2 and lentivirus vector carrying *SKIL* gene (shSKIL#2+ox-SKIL), SKIL expression was increased compared to control cells transfected with control shRNA (shNC). **(E)** Cell viability was decreased in NCI-H520 cells transfected with shSKIL#2 and empty lentivirus vector (shSKIL#2+ox-vector) compared to control cells (shNC); cell viability was increased in NCI-H520 cells transfected with both shSKIL#2 and lentivirus vector carrying *SKIL* gene (shSKIL#2+ox-SKIL), compared to control cells transfected with control shRNA (shNC). **(F)** Cell migration and invasion were decreased in NCI-H520 cells transfected with shSKIL#2 and control lentivirus vector (shSKIL#2+ox-vector), compared to control cells transfected with control shRNA (shNC); cell migration and invasion were increased in NCI-H520 cells transfected with both shSKIL#2 and lentivirus vector carrying *SKIL* gene (shSKIL#2+ox-SKIL), compared to control cells transfected with control shRNA (shNC). **(G)** E-cadherin expression was increased and vimentin expression was decreased in NCI-H520 cells transfected with shSKIL#2 and control lentivirus vector (shSKIL#2+ox-vector), compared to control cells transfected with control shRNA (shNC); E-cadherin expression was similar and vimentin expression was decreased in NCI-H520 cells transfected with both shSKIL#2 and lentivirus vector carrying *SKIL* gene (shSKIL#2+ox-SKIL), compared to control cells transfected with control shRNA (shNC). **P* < 0.05, ***P* < 0.01, ****P* < 0.001. Experiments were performed in triplicate.

**Figure S4 TAZ silencing promoted T cell infiltration through activation of STING pathway.** TAZ-silenced M109 mice lung cancer cell line was injected subcutaneously into BALB/c mice, and on day 21, mice were euthanized and tumor blocks were collected. Flow cytometry analysis showed significantly more **(A & B)** total T cells and **(C & D)** cytotoxic T cells in TAZ-silenced tumor block compared to control. qPCR measurement and western blot showed **(E – H)** significantly increased levels of chemokines (CXCL10, CCL5, IFN-β) and **(I & J)** elevated levels of STING, p-TBK1 and p-IRF3 in TAZ-silenced CALU-3, NCI-H520, M109 cell lines and tumor blocks with treatment of cGAMP, a STING pathway agonist. **P* < 0.05, ***P* < 0.01. Experiments were performed in triplicate.

**Figure S5 SKIL silencing in CALU-3 cells using shSKIL showed no significant influence on the expression of chemokines induced by Imiquimod or poly (I:C).**

**Figure S6 Densitometric analysis on western blot results of indicated figure.**

**Figure S7 Densitometric analysis on western blot results of indicated figure.**
