# Supplementary figures and images for: SKIL facilitates tumorigenesis and immune escape of NSCLC via upregulating TAZ/autophagy axis
Source: Cell Death Dis. 2020 Dec 2;11(12):1028. doi: 10.1038/s41419-020-03200-7 (PMC7710697; doi:10.1038/s41419-020-03200-7)

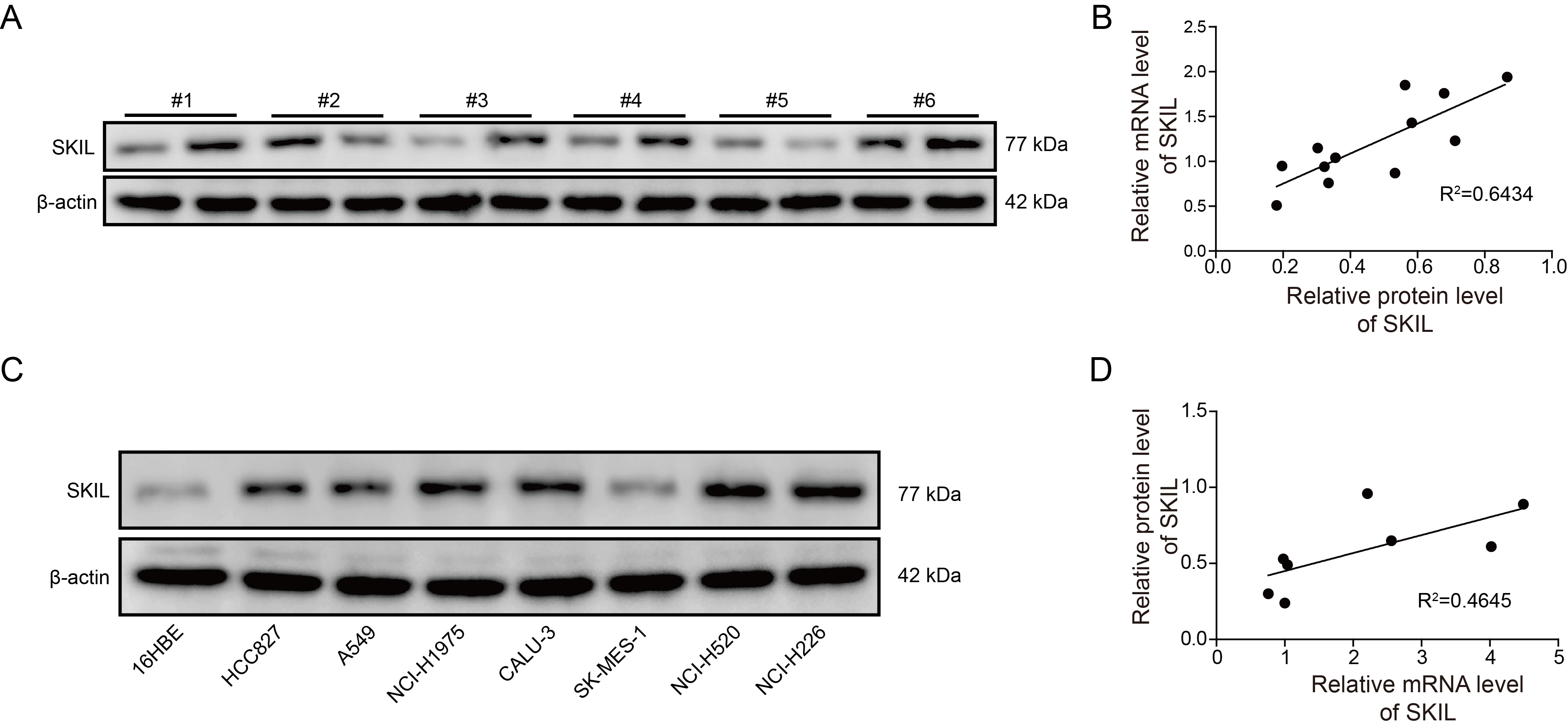

Supplement: Supplementary file 2 — FigS1 [file 41419_2020_3200_MOESM2_ESM.tif]

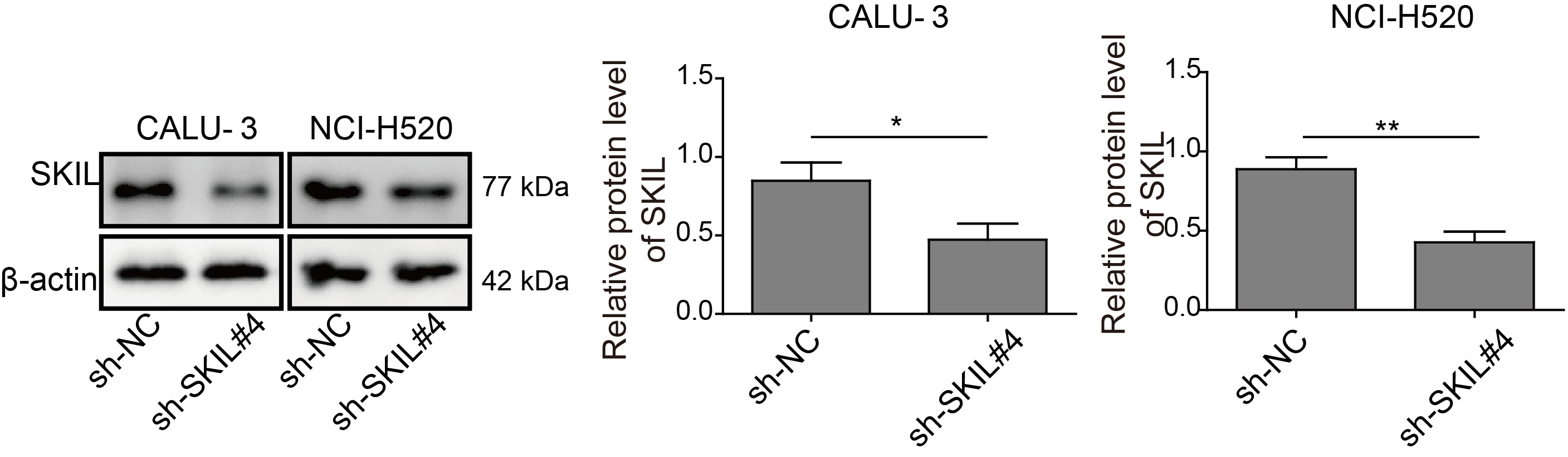

Supplement: Supplementary file 3 — FigS1 [file 41419_2020_3200_MOESM3_ESM.tif]

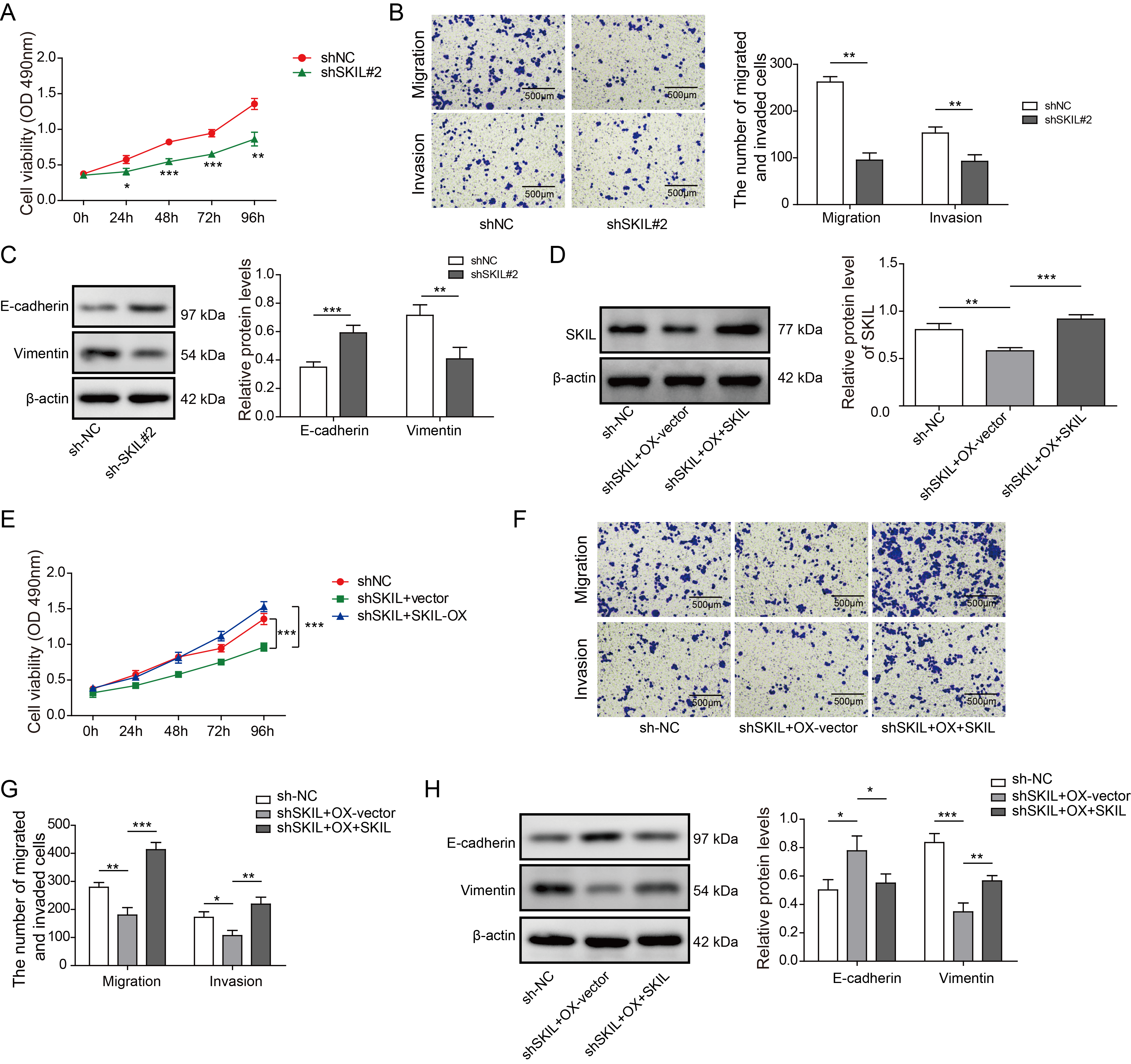

Supplement: Supplementary file 4 — FigS3 [file 41419_2020_3200_MOESM4_ESM.tif]

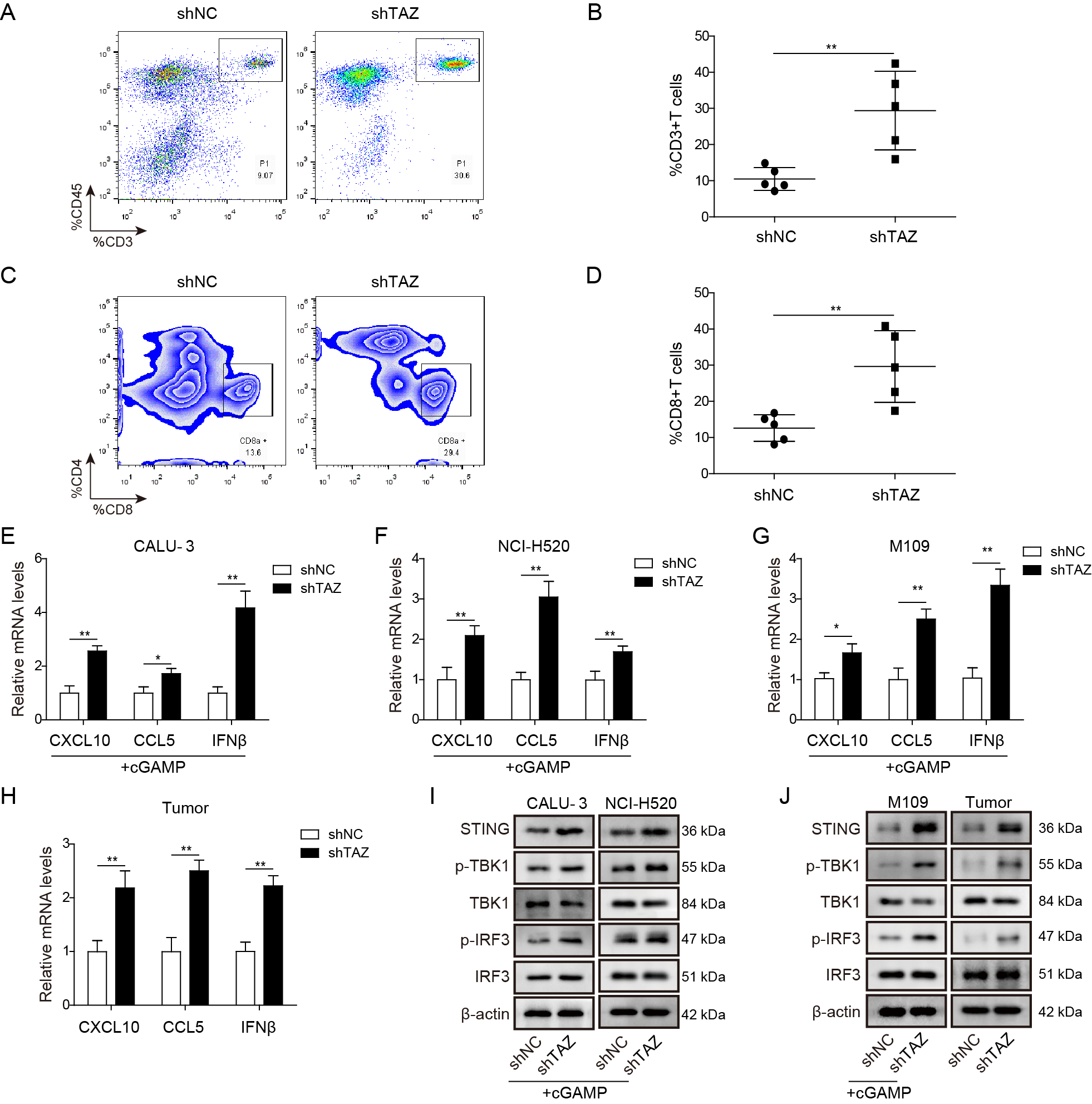

Supplement: Supplementary file 5 — FigS4 [file 41419_2020_3200_MOESM5_ESM.tif]

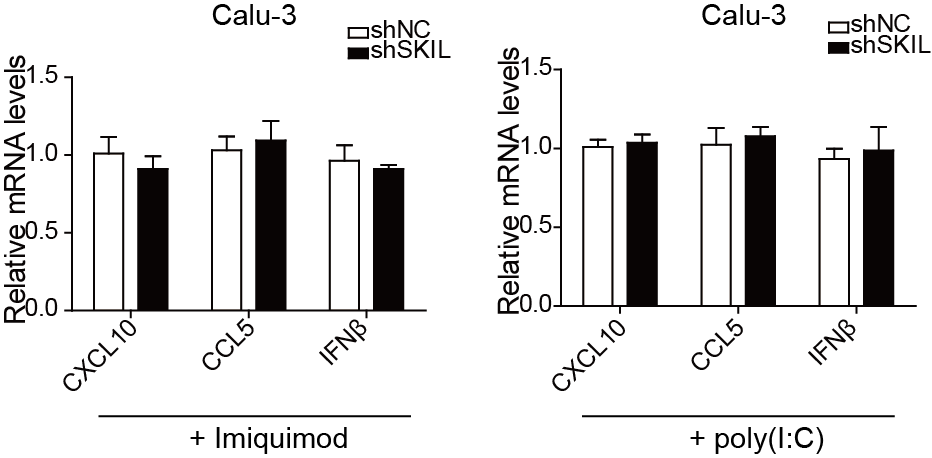

Supplement: Supplementary file 6 — FigS5 [file 41419_2020_3200_MOESM6_ESM.tif]

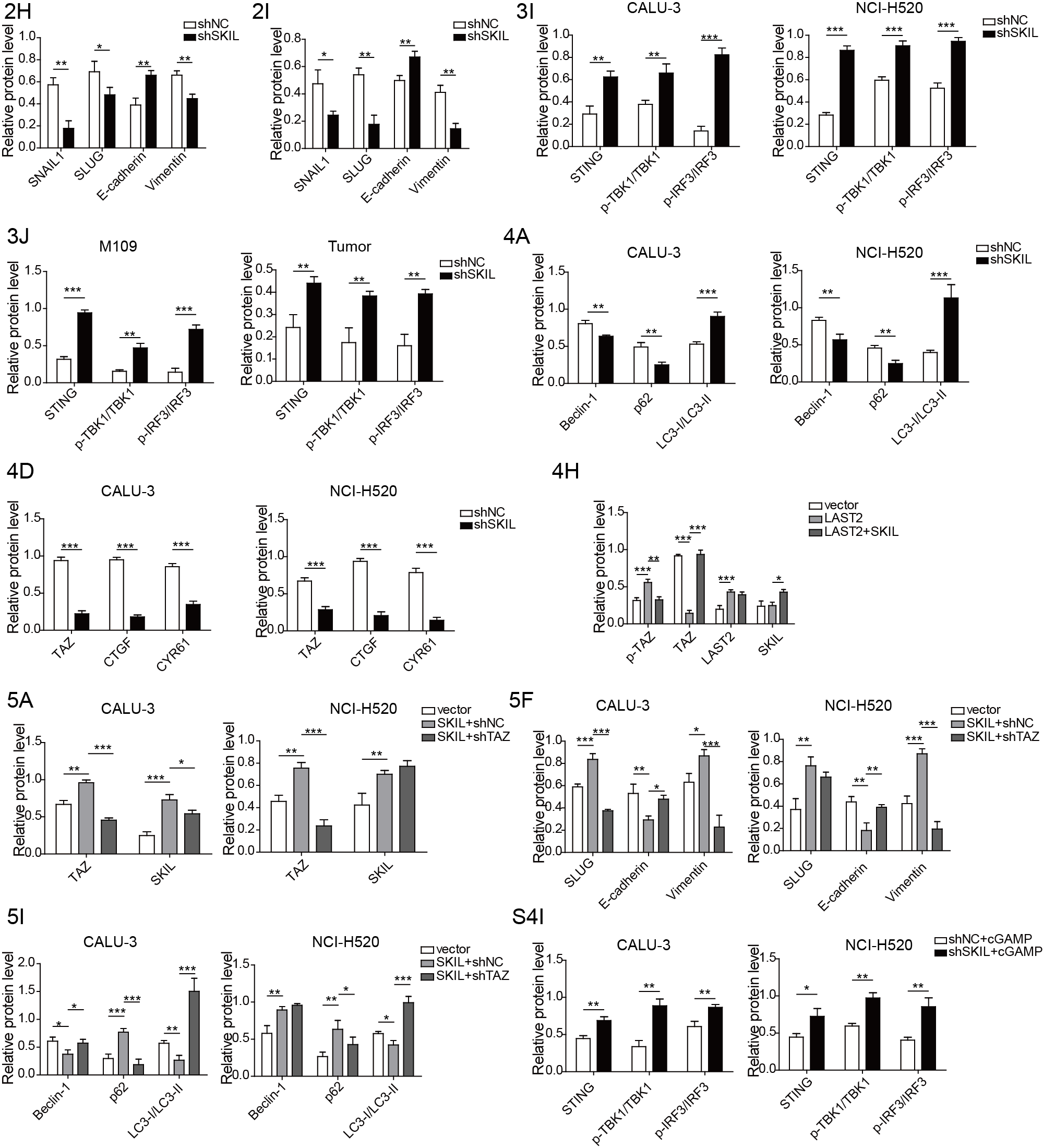

Supplement: Supplementary file 7 — FigS6 [file 41419_2020_3200_MOESM7_ESM.tif]

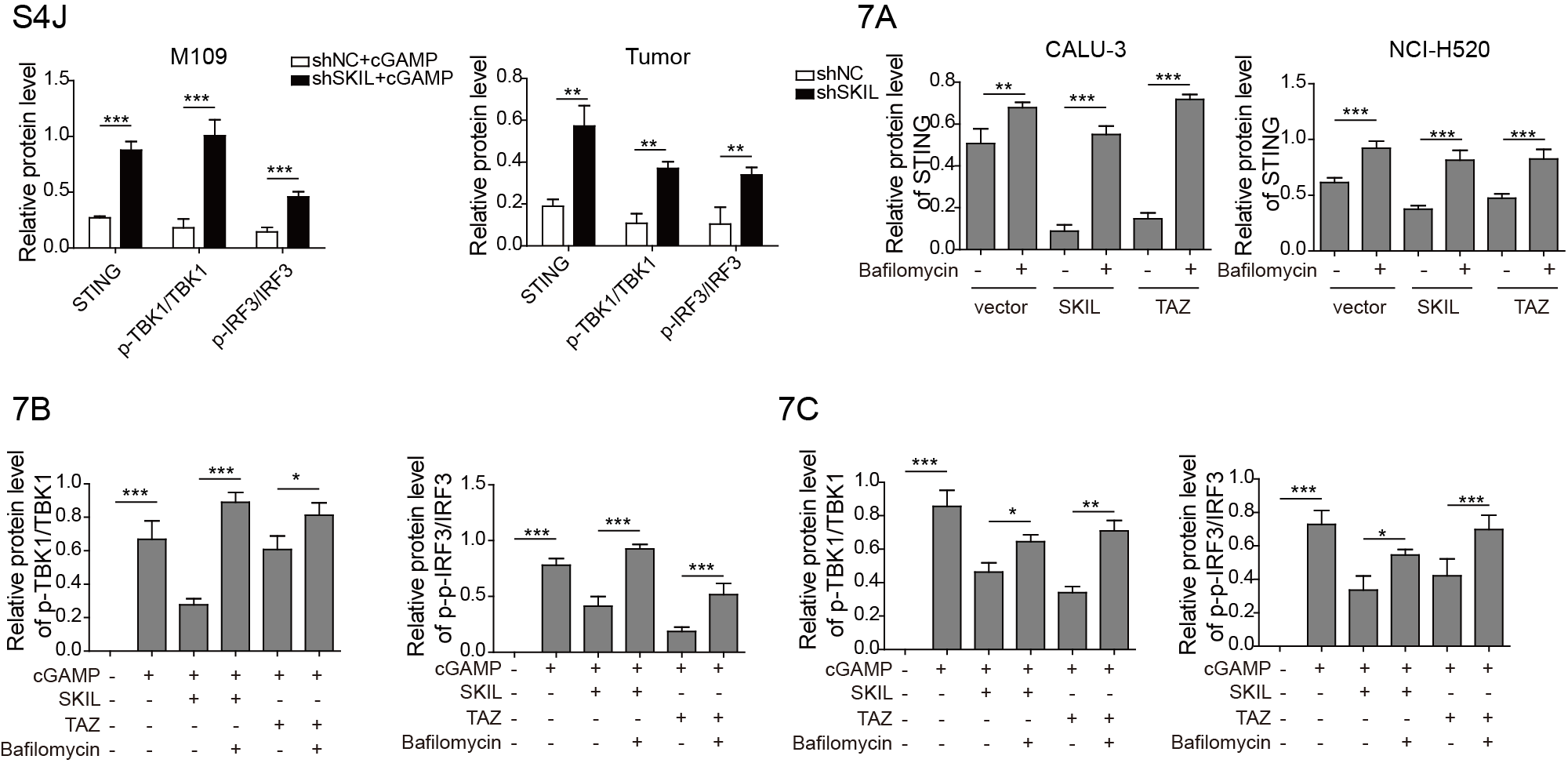

Supplement: Supplementary file 8 — FigS7 [file 41419_2020_3200_MOESM8_ESM.tif]
